# A note on the probability distribution function of the surface electromyogram signal^☆^

**DOI:** 10.1016/j.brainresbull.2012.09.012

**Published:** 2013-01

**Authors:** Kianoush Nazarpour, Ali H. Al-Timemy, Guido Bugmann, Andrew Jackson

**Affiliations:** a Institute of Neuroscience, Newcastle University, United Kingdom; b Centre for Robotics and Neural Systems (CRNS), Plymouth University, United Kingdom

**Keywords:** Electromyogram signal, Higher order statistics, Probability distribution function

## Abstract

The probability density function (PDF) of the surface electromyogram (EMG) signals has been modelled with Gaussian and Laplacian distribution functions. However, a general consensus upon the PDF of the EMG signals is yet to be reached, because not only are there several biological factors that can influence this distribution function, but also different analysis techniques can lead to contradicting results. Here, we recorded the EMG signal at different isometric muscle contraction levels and characterised the probability distribution of the surface EMG signal with two statistical measures: bicoherence and kurtosis. Bicoherence analysis did not help to infer the PDF of measured EMG signals. In contrast, with kurtosis analysis we demonstrated that the EMG PDF at isometric, non-fatiguing, low contraction levels is super-Gaussian. Moreover, kurtosis analysis showed that as the contraction force increases the surface EMG PDF tends to a Gaussian distribution.

## Highlights

► We recorded surface EMG signals with a biofeedback setup at 7 different contraction levels. ► We estimated the PDF, kurtosis and bicoherence index of the measured signals. ► We show that the EMG PDF at low contraction levels is super-Gaussian. ► At higher contraction forces, the EMG PDF tends to a Gaussian distribution.

## 1 Introduction

A surface electromyogram (sEMG) signal is the electrical manifestation of the neuromuscular activity and is recorded non-invasively from the surface of the skin (Hogan and Mann, 1980; deLuca, 1979). The sEMG signal has been extensively used for estimation and interpretation of the neural drive to muscles (Merletti et al., 1999), extraction of a voluntary command signal for control of prosthetic devices for individuals suffering from limb amputation (Hefftner and Jaros, 1988; Park and Meek, 1995; Huang et al., 2005), and in biofeedback experiments in which the subjects learn to change patterns of voluntary muscle contraction (Ince et al., 1984; Radhakrishnan et al., 2008; Bloom et al., 2010; Nazarpour et al., 2012).

Conventionally in the prosthetic control applications after a pre-processing stage, several features are extracted from the EMGs and a decoder is trained to recognize different patterns of muscle activity. Various features in time and frequency domains have been introduced for this purpose – for a review see Micera et al. (2010). Higher order statistics (HOS) (Mendel, 1991) of the EMGs have also proved effective in movement classification (Nazarpour et al., 2005b, 2007). The merit of such HOS-based approaches lies in their capability of capturing the skewness and pickedness (and other higher order statistics) details of the EMG PDF that are ignored when the EMG is assumed to be Gaussian process and consequently the first- and the second-order moments and cumulants (i.e., mean, correlation, and variance) and their spectral representations are analysed only.

Despite the success of HOS-based methods, there is not yet a general consensus upon the PDF of the EMG signals to justify the application of these statistics. For instance in Ref. (Roesler, 1974), it was shown that a Gaussian density function can precisely model the EMG PDF at various contraction strengths. Parker et al. (1977) also showed that EMG recorded at reasonably low contraction levels can be modelled with a Gaussian process. In contrast Hunter et al. (1987) and Bilodeau et al. (1997) used kurtosis analysis and reported that during low intensity isometric contractions the PDF of the sEMG signal is more peaked near zero than a Gaussian distribution. They also reported that there was tendency for the kurtosis values to decrease with increasing contraction level implying that the EMG PDF becomes closer to a Gaussian distribution since the third- and the fourth-order statistics of a pure Gaussian process are equal to zero. Clancy and Hogan (1999) also showed that the PDF of the EMGs recorded during constant-angle, constant-force, and non-fatiguing contractions falls between the Gaussian and the Laplacian densities. Negentropy analysis of the EMG signals (Nazarpour et al., 2005a; Naik et al., 2011) showed that the non-Gaussianity level of the EMG signal depends on the muscular contraction level such that the increment in the contraction level shifts the EMG PDFs towards the Gaussian distribution.

Kaplanis et al. (2000) explored the EMG PDF by investigating the bicoherence index of the EMG measurement. However, they arrived at the conflicting result that the EMG signal is more non-Gaussian at low and high levels of force while being in its maximum Gaussianity at the mid-level (50 %) of maximum voluntary contraction (MVC). Recently in Hussain et al. (2009), the bicoherence analysis was used to test the Gaussianity of the EMG signals and it was shown that the EMG becomes less Gaussian with increased walking speed force (increase in mean voluntary contraction).

In this paper, we revisited this problem and investigated the suitability of the bicoherence of the sEMG signal for characterization of the non-Gaussianity level of the sEMG signals for different levels of muscular activity.

## 2 Method

### 2.1 Participants

Four right-handed subjects (two female; mean age: 26 ± 5 years) participated in the study. They were free of any history of neurological or motor disorders and gave informed consent. The study was approved by the local ethics committee at the Institute of Neuroscience, Newcastle University.

### 2.2 Experimental setup

Subjects controlled a myoelectric cursor (Radhakrishnan et al., 2008; Nazarpour et al., 2012) by making isometric contractions of a single right upper-limb muscle. We recorded surface EMG signals (Bio-logic disposable snap electrodes, Natus Medical Inc.) from Abductor Pollicis Brevis (APB: abducts the thumb) and Flexor Carpi Radialis (FCR: flexes the wrist) muscles. Subjects completed two independent runs of the experiment (6 blocks), one for each muscle as the controlling effector. The skin was cleansed with alcohol beforehand and the electrode locations were chosen to maximize the quality of recording. EMG measurements were amplified (gain 1–10 K) and high-pass filtered at 30 Hz (Neurolog NL824, Digitimer) before sampling at 10 kHz (PCI-6071E, National Instruments). The hand was restrained in an open, pronated posture inside a glove fixed to a horizontal board and the forearm was strapped to the arm-rest of the chair. At the start of the experiment, subjects were informed of the general structure of the experiment.

In the first (of six) block we asked the subjects to produce five contractions with their maximum voluntary contraction level (MVC) for a period of two seconds (100% MVC). In the second block, we instructed the subjects to contract the muscle at a slightly lower level than in the first block. As will be mentioned later in the results section, subjects on average produced an activity of only about 50% MVC. They repeated the same procedure in the fifth and the sixth blocks. In these four blocks no visual feedback was provided.

At the start of the third block, subjects were instructed to produce comfortable levels of contraction of each muscle which they would be able to repeat many times without fatigue. This corresponded to approximately 5–10% of their maximum voluntary contraction level of that muscle. The true contraction levels were verified offline. In the third and fourth blocks (each of 100 trials), the subjects controlled the position of a myoelectric cursor along a 1D vertical task space. The control signal was computed every 13ms by smoothing (with a rectangular window) the preceding 500 ms of rectified EMG. Subjects initiated a trial by relaxing the controlling muscle to bring the cursor to a starting zone and remaining there for 250 ms after which a target appeared. The remainder of the trial was divided into two fixed periods of 1 and 3 s, designated movement and hold periods. Auditory tones cued the start of the movement and hold periods. At the end of each trial, subjects received a score reflecting the proportion of the hold period that the cursor was inside the target and were instructed to maximize this score. In each trial, a target was presented in one of five possible positions along the vertical axis; the order of the targets was pseudo-random. Targets one to five could be reached by producing an activity (with thumb abduction or wrist flexion whichever instructed) as large as one to five times comfortable contraction level, respectively. In approximately 2% of trials, subject could not hold the cursor inside the target area. We excluded these trials from analysis. Visual feedback was available throughout blocks 3 and 4.

### 2.3 Offline verification of contraction levels

In contrast to earlier studies in which the EMG signals were recorded at fixed contraction level e.g. 25%, 50% MVC, we allowed the subjects to determine their comfortable contraction level required to hold the cursor in target 1. These comfortable contraction levels were different across subjects and muscles. We determined the actual contraction percentage by calculating the average mean absolute value (MAV) of EMG during the hold period for each target (20 presentations). After adjusting for the amplifier gain, we normalized these MAVs to the MVC activity (averaged over the 5 trials) (in each subject and for each muscle) with

(1) $$\%\;\mathrm{of}\;\mathrm{MVC}=\frac{\frac{1}{20}\sum_{i=1}^{20}{\mathrm{MAV}}_{i}}{\frac{1}{5}\sum_{j=1}^{5}{\mathrm{MVA}}_{j}\;\mathrm{of}\;100\%\;\mathrm{MVC}}$$

### 2.4 Bicoherence analysis

A frequency-domain measure of the third-order cumulant $C_{3}^{\text{x}}(m,n)$ is the bispectrum (Hinich, 1982) and is calculated by taking a two-dimensional discrete-time Fourier transform from $C_{3}^{\text{x}}(m,n)$ with

(2) $$B^{\text{x}}(w_{1},w_{2})=\sum_{m,n=-\infty }^{+\infty }C_{3}^{\text{x}}(m,n)e^{-j(w_{1}m,w_{2}n)}.$$

The normalized bispectrum is called bicoherence and is computed with

(3) $${\mathrm{Bic}}^{\text{x}}(w_{1},w_{2})=\frac{B^{\text{x}}(w_{1},w_{2})}{P^{\text{x}}(w_{1})P^{\text{x}}(w_{2})P^{\text{x}}(w_{1}+w_{2})}$$

where $P^{\text{x}}(w)$ denotes the power spectrum of **x** at frequency w. Bicoherence can be used to measure the skewness of a random process (Mendel, 1991). For that purpose, a test of Gaussianity was defined in (Hinich, 1982) by the mean bicoherence power

(4) $$S^{\text{x}}=\sum_{w_{1},w_{2}}|{\mathrm{Bic}}^{\text{x}}(w_{1},w_{2}){|}^{2}$$

and is compared with a central chi-squared distribution; in essence if ${\mathrm{Bic}}^{\text{x}}(w_{1},w_{2})$ is zero then the *S*^**x**^ statistic is a central chi-squared distributed random variable with two degrees of freedom – see (Hinich, 1982) for mathematical proof.

### 2.5 Kurtosis analysis

The kurtosis of a random variable is computed by dividing its fourth cumulant by the square of its second cumulant. Sample kurtosis for a univariate random process “**x**” can be estimated with

(5) $$kurt_{\text{x}}=\frac{E\{{\text{x}}^{4}\}}{E{\left\{{\text{x}}^{2}\right\}}^{2}}-3$$

where *E*{ . } denotes the statistical expectation operator. Kurtosis measures the peakedness of a PDF.

A MATLAB R14-based graphical user interface linked to Cogent (2000) was developed to control this experiment. All data analysis was carried out in MATLAB.

## 3 Results

**Fig. 1:**
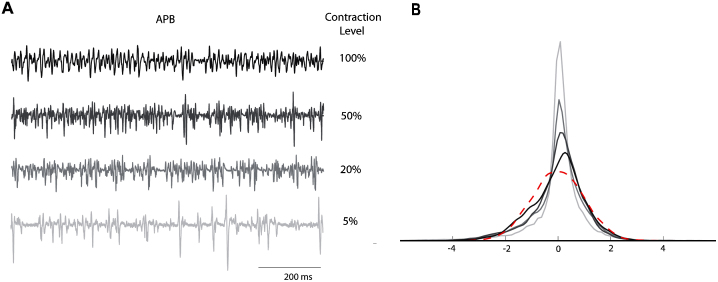
(A) Representative example of raw EMG data recorded from APB muscle at different percentages of MVC and the corresponding PDFs in (B) where the PDF of a Gaussian distributed variable of the same length is depicted by a dashed curve. At lower contraction levels the PDF of the EMG signal is more peaked at zero. Note that for clear presentation we standardized the EMG recordings and hence the absolute scale of the vertical axes in (A) and (B) is arbitrary.

Fig. 1A shows a representative set of raw EMG recorded from APB in one subject for different contraction levels. Fig. 1B depicts the probability distribution functions that are estimated using the kernel smoothing method (Parzen, 1994) with Gaussian kernels. For comparison purposes, the PDF of a random variable of the same length drawn from a normal distribution is also depicted. Note that in Fig. 1, only for clarity of presentation, all signals are standardized to zero mean and unit variance. This operation has no effect on the higher order statistics of these signals but renders the vertical axes in Fig. 1A and B arbitrary.

**Fig. 2:**
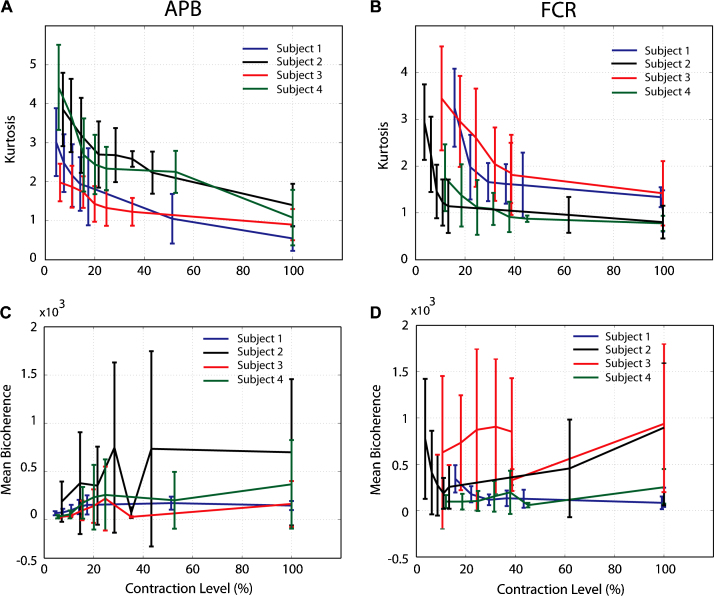
(A, B) present the averaged estimated kurtosis of the EMG signals in a range of contraction level from four subjects; bars show the standard deviations. Clearly, with an increase in the contraction level the kurtosis values decreases. (C, D) depict the averaged values of the estimated mean bicoherence indices for the measured EMG from the same muscles. No clear trend for modulation of mean bicoherence index with the contraction.

Fig. 2A and B displays the computed mean of kurtosis values of the APB and FCR muscle activity relative to the percentage of the MVC activity for individual subjects. Importantly, the mean of kurtosis reduced for all subjects and in both muscles when the contraction level increased reflecting a shift from a non-Gaussian distribution to a more Gaussian-like distribution. A two-way (muscle and contraction level) ANOVA test confirmed the main effect of contraction level (repeated measures, *F*_6,18_ = 87.37, *p* < 0.001, *n* = 4). The main effect of muscle was not significant (*F*_1,3_ = 0.927, *p* = 0.40, *n* = 4). Fig. 2C and D shows the mean bicoherence indices computed for APB and FCR muscles for different force levels. In contrast to (Kaplanis et al., 2000; Hussain et al., 2009), we did not observe any consistent trend in mean bicoherence index relative to contraction level (*F*_6,18_ = 2.51, *p* > 0.05, *n* = 4) in either muscle.

## 4 Concluding remarks

By analysis of the kurtosis of the EMG signals we showed that at low contraction levels, EMG PDFs are more peaked at zero. When the force level increases, the EMG PDF tends to a more bell-shaped Gaussian distribution. Related physiological work have shown that increasing the force level will not only increase the rate of the already firing motor units (temporal recruitment), but also recruits more motor units of same or other types (Fuglevand et al., 1993). The central limit theorem (CLT) predicts if sufficiently large number of (independent) motor units fire, the signal recorded from the surface of the skin will be approximately normally distributed. Our results are consistent with the predictions of the CLT.

Several earlier studies show that the sEMG signal irrespective of the contraction force level exhibits a symmetric distribution function that leads to small skewness $C_{3}^{\text{x}}(m,n)$ values (see (Nazarpour et al., 2007) and reference therein). Authors of (Kaplanis et al., 2000) and (Hussain et al., 2009) overlooked the fact that the so-called bispectrum index-based Gaussianity test (Hinich, 1982) only quantifies the skewness of a probability. Therefore, the Gaussianity test in Hinich (1982) may only be used to reject the Gaussianity null hypothesis. If the bispectrum index is zero, the Gaussianity of the process may not be inferred since fourth and higher-order cumulants and polyspectra would not necessarily be zero (Mendel, 1991). For instance, if a signal has a Laplacian distribution, the bispectrum and all the odd-ordered polyspectra are zero, however, the even-ordered statistics (e.g. kurtosis) or polyspectra (e.g. the trispectrum) will not identically be equal to zero.

In contrast to (Bilodeau et al., 1997; Clancy and Hogan, 1999; Nazarpour et al., 2007) in which the EMG signals were recorded at fixed percentages of the maximum contraction level (MVC), we deliberately recorded the EMGs in a more flexible range of the force levels so that we can quantify the PDF of the sEMG signals in a broader range of force levels.

We characterized the PDF of the EMG signals at different contraction levels in two muscles. However, the choice of the muscle should not influence our main results significantly. Sanger (2007) and also we in Nazarpour et al. (2007) examined the PDF of Biceps and Triceps muscles at difference contraction levels and arrived at a comparable result that a Laplacian distribution is more suitable for EMG PDF modelling measured at low contraction levels. However, not only other biomechanical factors, such as contraction speed and isometricity of contraction, but also several anatomical, e.g., number of active motor units, size of the motor units, the spatial distribution of motor units and physiological factors (neural disorder and fatigue) can influence the shape of the EMG PDF. In addition, the measurement noise (e.g. crosstalk and electronic interferences) can change the PDF of the recorded signals. These factors might explain the lack of consensus upon the EMG PDF in literature.

The demonstration that the PDF of the sEMG signal recorded at low forces is closer to a Laplacian distribution may have significance for prosthesis control or biofeedback experiments since this could form a flexible substrate for developing novel mathematical tools tailored for super-Gaussian processes such as higher order statistics. For instance, Sanger (2007) developed a Bayesian algorithm to predict the envelope of the EMG signals and showed that by assuming an exponential density (half-Laplacian) for the sEMG signal the output of a Bayesian filter follows the rapid changes in the EMG amplitude much faster than the conventional linear approaches. Nevertheless, successful use the HOS of surface EMG for prosthesis for control and biofeedback depends on the reliability of the algorithms that estimate these statistics accurately. Our current work includes developing robust and efficient algorithms to estimate recursively the sample kurtosis value in real-time.
